# Teenagers and Adolescents with Hemophilia–Need for a Specific Approach

**DOI:** 10.3390/jcm13175121

**Published:** 2024-08-29

**Authors:** Christoph Königs, Jayashree Motwani, Víctor Jiménez-Yuste, Jan Blatný

**Affiliations:** 1 Goethe University, University Hospital, Department of Paediatrics and Adolescent Medicine, Clinical and Molecular Haemostasis, 60590 Frankfurt, Germany; 2 Department of Paediatric Haematology, Birmingham Children’s Hospital, Birmingham B4 6NH, UK; jayashree.motwani@nhs.net; 3 Hematology Department, Hospital Universitario La Paz-IdiPaz, Autónoma University, 28046 Madrid, Spain; victor.jimenez@uam.es; 4 Department of Paediatric Hematology, University Hospital and Masaryk University Brno, 601 77 Brno, Czech Republic; jblatny@icloud.com

**Keywords:** adolescence, adolescent, hemophilia, management

## Abstract

Adolescents with hemophilia are a patient population with special requirements, having to manage their condition alongside the typical challenges of adolescence. Given the psychosocial impact of hemophilia and a desire to fit in with non-hemophilic peers, they may perceive treatment as more of a burden than a benefit. This can result in low adherence and a high risk of hemophilia-related complications. Hemophilia management has changed over time. To best inform shared decision-making with adolescent patients and their families, healthcare professionals must consider all the currently available evidence, highlighting treatment benefits as appropriate. They should also appreciate the requirements of all adolescents affected by hemophilia, including individuals with non-severe disease and girls/women. We discuss specific issues relating to the management of adolescents with hemophilia: prevention and management of bleeds, treatment adherence, joint health and physical activity, and other health-related issues. A multidisciplinary approach is advocated, and the potential role of digital technology in helping to equip patients with self-management skills to fully engage with treatment is considered. Currently, available hemophilia management generally enables adolescents with hemophilia to lead normal lives, participating in physical activities while maintaining good joint health. However, more work is required to help address both actual and perceived limitations.

## 1. Introduction

The World Health Organization defines adolescence as the stage of life between 10 and 19 years of age [1]. However, this definition is not uniformly recognized, suggesting a lack of understanding of issues applicable to this age group. For example, as individuals who have a chronic condition progress through adolescence, they will become less dependent on parental/caregiver support in a different way from those without a chronic condition and gradually gain an increasing appreciation of the challenges associated with living with their condition and the medical care required to manage this. Based on the current understanding of growth patterns and social role transitions, some argue that adolescence now spans a greater time period than ever before [2]. Levels of maturity can also differ between individuals, as well as through global and local variations in family situations. As per the clinical experience of the authors of this article, and in line with published viewpoints [2], for this review, we have defined adolescence as the period of life ranging from 10–24 years, which includes young adults.

Adolescence is a challenging transition period for those with a chronic condition, potentially impacting care [3,4,5]. Adolescents with hemophilia represent a population with special requirements. They are at high risk of developing hemophilia-related complications due to issues associated with low adherence [6], the psychosocial impact of the disease [7], and the burning desire to fit in with their peers; after many years of successful treatment, some may perceive their treatment as no longer necessary, and more of a burden than benefit [6,7]. Patients with hemophilia must face the typical challenges of adolescence alongside challenges specific to hemophilia [8]. 

Hemophilia management has changed over time, and healthcare professionals must consider all available evidence to best inform the shared decisions made with adolescent patients and their families, highlighting the benefits of continuing effective treatment received during childhood, as appropriate. The aim of this manuscript is to identify the specific issues related to the management of adolescents who have been diagnosed with hemophilia A or B. By providing suggestions, as well as potential answers, as to how these issues may be addressed (Table 1), the intention is to improve care by empowering healthcare professionals to feel better equipped to manage this particular patient population. The transition from pediatric to adult hemophilia health centers is also an important aspect of adolescence; however, this topic is considered in more detail elsewhere [3].

## 2. Prevention and Management of Bleeds

### 2.1. Prophylaxis and Personalized Therapy

To prevent bleeding, long-term prophylaxis is the standard of care for individuals with severe hemophilia and moderate hemophilia with a severe phenotype [9]. Although there are regional/country-specific differences, healthcare professionals in many regions currently have access to a range of treatments, with new options becoming available and even more on the horizon [10]. Although the specific available treatments vary according to the region/country in which patients are being treated, generally, there is now a greater number of options to consider when managing adolescent patients with hemophilia. 

With the range of potential treatment options, healthcare professionals are able to offer more individualized therapy than in the past. For adolescents, changes in body weight and composition due to rapid growth during this period of life will most likely influence the individualization of therapy. In addition, adolescence heralds other changes, including lifestyle, which can affect expectations relating to therapy and the basis on which therapeutic decisions are made. A new therapeutic strategy for healthcare professionals to consider for patients with hemophilia, including adolescent patients, is taking a tailored approach to developing a personalized prophylactic treatment regimen with whatever treatment option has been chosen. This personalized treatment can extend beyond individualized treatment via pharmacokinetic parameters [11] and bleeding phenotype to also take into account a patient’s goals and lifestyle, which will differ for each patient [9]. Therefore, personalized or ‘precision’ medicine plays a role in the treatment of hemophilia. While there has been much clinical focus on those with severe hemophilia [12], it is important to appreciate the requirements of individuals with hemophilia who are affected by non-severe disease, and also include women and girls. However, specific issues relating to women and girls are beyond the scope of this article but are considered elsewhere [13,14]. 

### 2.2. Dealing with Bleeds during Adolescence

A hallmark of hemophilia A and B is bleeding, which may affect joint and muscle health [9,15]. However, it could be argued that this is a particularly pertinent issue for adolescents with hemophilia. The frequency of bleeding events can increase during the physical strains of adolescence [16]; biological changes in the muscles and joints during this period of growth may influence bleed management [9]. Conversely, due to the success of long-term prophylaxis, many adolescents may not have experienced a bleeding event for many years or have a low burden of bleeds [5,7]. With limited experience with bleeding events, adolescents with hemophilia may begin to question the necessity and benefits of prophylaxis, potentially becoming non-adherent [5,6] due to the burden of treatment. In a mixed methods study conducted in Germany, interviewing patients with hemophilia A or B aged 15–29 years revealed a ‘high degree of satisfaction’ and ‘treatment success’ [17]. However, some adolescent patients said they would prefer ‘fewer injections’ [17]. This can, in part, lead to low treatment adherence, which will be discussed in more detail in a subsequent section.

Patients may not always recognize when a bleed occurs; this can lead to a bleeding event being unnoticed or a delay in addressing it [5,7]. Alternatively, in situations where they may be reluctant to interrupt an ongoing activity, adolescents may not want to acknowledge a bleed. In contrast, a bleed can be overestimated, as pain does not always signify a bleed [18,19].

As opposed to the previous generation, who experienced more bleeds with less available or less advanced treatment, the effectiveness of current treatments has meant that bleeds occur much less often than in the past. As a result, current generations of patients receiving these (prophylactic) therapies may be relatively unfamiliar with the symptoms of bleeds and have less direct experience with the benefits of their treatment [4,5,7]. In fact, younger patients may be growing up without gaining the necessary skills to recognize and treat bleeds. This lack of recognition can lead to delays in presentation [5,9]. For example, diagnosis of bleeds in the psoas muscles may be delayed due to presenting symptoms [15]; it could be incorrectly regarded as a torn muscle following sport. Therefore, it is very important that bleed recognition and early treatment are revisited regularly with adolescent patients [5]. In relation to this, while the importance of training from healthcare providers should be emphasized, it should also be appreciated that as younger physicians may have limited experience in recognizing and treating bleeds, physician awareness should also be considered [20]. With appropriate physician awareness and patient education tailored towards changes during adolescence and taking into account, for example, levels of sporting participation (Section 4.2) and possible complications associated with sexual activity (Section 5.3), bleed recognition and management can be improved.

### 2.3. Promoting Self-Care

The responsibility for preventing and managing bleeds understandably falls on the parents and/or caregivers of children with hemophilia [7,9]. Parents of children with bleeding disorders have been found to be generally pleased with the care provided by specialist teams, with a subsequent good understanding of their child’s condition and confidence in dealing with this [21]. Nevertheless, the condition can have an emotional impact and there is a need for parental support to be regularly assessed [21]. However, as patients progress through adolescence, they need to learn how to perform injections independently and subsequent complex self-management skills, including bleed management [22]. This involves taking more, and subsequently full, responsibility for one’s own condition and relying less on the support of others [6]. Therefore, ensuring that adolescents with hemophilia are equipped with the knowledge and tools required for self-advocacy is of paramount importance. To promote self-care management, adolescents with hemophilia should ideally receive education and training from hemophilia healthcare professionals [9]. The previously mentioned study noted that some adolescents responded positively to the opportunity of being able to practice certain self-management skills together with their peers [17]. Patients learn how to perform different self-management tasks at different stages of adolescence, with more complex skills, such as bleed management, typically learned in late adolescence [23]. It is recommended to start learning how to perform injections independently before the onset of puberty. Adolescents should be encouraged to have progressively increased involvement in their own care over time, building knowledge and confidence in relation to managing hemophilia. Healthcare professionals should continue to educate patients throughout adolescence, including the potential use of digital communication tools to deliver education [23]. However, for adolescents to assume full responsibility for their own health, it is critical that their parents and/or caregivers give them the space to do so. Parents should avoid over-protecting their children and instead should support them to prevent and manage their bleeds by themselves [7]. Healthcare professionals could facilitate this transfer of responsibility by arranging healthcare consultations with patients without their parents or caregivers present [7]. However, psychosocial support of caregivers should also be available when education in self-management is provided [7].

## 3. Treatment Adherence

### 3.1. Recognizing Challenges Associated with Adolescence

Treatment adherence in patients with chronic conditions is often low [24]. Consistent with other chronic conditions, treatment adherence can also be an issue in hemophilia. In a global study involving centers treating people with hemophilia, adherence to all types of prophylaxis was highest in the youngest age group. In fact, 59% of patients aged 0–12 years old achieved over a 90% level of adherence, whereas in the 13–18 age group, only 13% achieved this level of adherence, with 6% and 17% achieving over 90% adherence in the 19–28 age group and the group over 28 years of age, respectively [25]. In hemophilia, low treatment adherence can increase the risk of bleeding and arthropathy [9], while adolescents and young adults who adhere to therapy have a lower likelihood of experiencing chronic pain [26].

As patients transition from childhood to adolescence, treatment adherence may wane as the parental influence decreases and patients start becoming more responsible for their own healthcare [6]. Treatment compliance with prophylactic factor therapy often decreases when patients enter adolescence—adolescents tend to focus on the present and may lack awareness about the risk of long-term joint damage due to non-adherence if they have received successful prophylactic treatment since childhood [27]. Furthermore, immediate physical and social activities are typically prioritized over long-term health goals during adolescence [28]. Moreover, some adolescent patients have expressed concern about injections, documenting treatment, and the timing of appointments [17]. Additional barriers compromising treatment adherence include adolescents not wanting to appear different from their peers, the perceived negative impact of treatment on social participation, escaping from parental overprotection, low symptom burden, forgetfulness, lack of knowledge about hemophilia, inability to recognize and act on bleeds, and disease denial [7]. Weighing up the risk associated with certain activities [6], some adolescents may consider that an activity is safe without prophylaxis.

### 3.2. Overcoming Barriers

Responding to a survey on policies in changing prophylaxis regimens in adolescents with severe hemophilia, healthcare professionals stated that to overcome some of the barriers associated with adolescence, prophylactic treatment is often modified by selecting treatments with more flexible dosing intervals [29]. This makes treatment adherence a more realistic possibility and can thus improve outcomes. In part due to the availability of new treatments, which have more convenient dosing regimens that are less intrusive on daily life and/or may provide higher levels of protection [12], healthcare professionals now have more options to ‘bargain’ with in discussions with adolescent patients, helping them to find the ‘right regimen for the right person’, taking into account patients’ individual needs. Discussing the goals adolescents wish to achieve, as well as other patient-related outcomes and how these may be facilitated by the product choice, can help patients appreciate the benefits of treatment beyond bleed protection in terms of enabling lifestyle choices. Products with extended half-lives can be used to tailor treatment by reducing injection frequency compared with standard half-life products and/or increasing levels of protection in line with expected activity [30]; these options can help to reduce treatment burden [12] or facilitate sporting participation [30], respectively. New factor therapy, ‘Ultra-Long factor VIII’, offers an option for high levels of bleed protection with weekly prophylaxis [31]. Non-factor therapy affords opportunities for decreased treatment burden via subcutaneous delivery [32]. In addition, exploring treatment options with patients themselves can aid a transition to shared decision-making with them rather than with their parents. It is also important for healthcare professionals to educate patients about prophylactic treatments prior to puberty whilst being amenable to adapting treatment to a patient’s evolving lifestyle [27]. Lastly, training patients to manage their own dosing schedules has previously been suggested to improve the acceptance and success of prophylaxis in adolescents [8]. 

Challenges to adherence may also be experienced by those who start prophylaxis during adolescence, such as patients with a moderate or mild phenotype or who have moved from a country where prophylaxis was previously unavailable. However, notably, post hoc clinical trial data have shown that patients with hemophilia aged 12–25 years who switched from on-demand to prophylactic therapy with extended half-life products remained on this therapy rather than returning to on-demand treatment [33].

## 4. Joint Health and Physical Activity

### 4.1. Joint Health

Joint damage is a common complication arising from bleeding in hemophilia. Early prophylaxis has been shown to preserve normal joint function with preserved joint health [34,35]. In many countries, the new treatments available can usually help children and adolescents grow up with more or less healthy joints; however, they also require a holistic approach with special considerations. Without appropriate treatment, patients with severe hemophilia will develop joint pain, swelling, and reduced range of motion later in life [15]. Joint damage starting during childhood may require treatment in subsequent decades, even for patients with non-severe hemophilia. Even for those who received appropriate treatment during childhood, a few joint bleeds can initiate a progressive process leading to synovitis and arthropathy, undoing the benefits of the many previous years of primary prophylaxis [36]. Point-of-care musculoskeletal ultrasound is practical and often available for clinical use to detect synovitis and joint damage [37]. This provides the ‘power of pictures’ to demonstrate early changes and help to educate and reinforce the value of prophylaxis for maintaining joint health.

### 4.2. Physical Activity

In the past, adolescents with hemophilia were actively discouraged from participating in sports due to the high risk of bleeding associated with physical activities [38]. As a result of the increased use of prophylactic treatments and with adolescents with hemophilia now mostly experiencing good joint health [39], it is now possible for people with hemophilia to participate in a range of sports [40] with an apparent lower risk of bleeding [41]. For all adolescents, engagement in sports has a range of benefits, both physical and somatic, and should be encouraged. In addition, for those with hemophilia, improved muscle strength may stabilize joints, decreasing the likelihood of bleeding complications [38]. Indeed, sporting participation among adolescents with hemophilia has greatly improved [38,42]. Yet, for patients, the desire to participate in physical activity may conflict with previously imposed parental or other restrictions. Indeed, although overall engagement in sports has increased, adolescents with hemophilia are often limited to only participating in non-contact sports, such as walking, running, and swimming [9,43]. However, due to the risk-prone nature of adolescents [7], many patients with hemophilia have the desire to play moderate- or high-impact sports, which include activities such as football or rugby [40,44]. Even with current prophylaxis, participation in high-impact sports remains problematic [43,44]. In fact, some patients have complained that ‘restrictions on the type of sports is the biggest limitation’ [17]. Despite some of the limitations around sports, it is still important to promote physical activity to adolescents with hemophilia. Regular exercise contributes to healthier joints, benefits quality of life and well-being, and reduces the risk of bleeding episodes [9,45,46]. Almost all patients interviewed as part of the previously mentioned study to understand the situation and needs of young people with hemophilia in Germany reported a benefit to having participated in sports [17]. When discussing physical exercise, adolescents may seek advice as to recommended activities/sports, the time at which these are best pursued, and appropriate prophylaxis. Although guidance is available from online sources, including the National Bleeding Disorders Foundation [47], target factor levels for various activities are purely expert opinion, and supporting data are required–recent publications indicate research is ongoing in this area [48,49]. In addition, general measures around stretching and preparations for taking part in physical activity need to be considered and physiotherapists can provide patients with information to help avoid sport-related injuries.

## 5. Other Health-Related Issues

### 5.1. General Health and Obesity

Patients with hemophilia often experience other health-related issues, especially during the period of adolescence. As previously mentioned, the increased use of prophylactic treatments in young people with hemophilia has enabled adolescents to engage more in physical activities. With increased participation in physical activities, the risk of developing obesity may have decreased. However, the prevalence of obesity is still higher in adolescent male patients with hemophilia compared with the general male population [50]. Investigations into the prevalence of overweight and obesity in young patients (median age of 13 years) with hemophilia A in Germany found that the prevalence of obesity was almost twice as high in children and teenagers with hemophilia compared with those without [51]. Obesity could be a particular problem for adolescents with hemophilia. A recent study in the USA has shown that obesity may be associated with worse joint health and function [52]. 

### 5.2. Quality of Life

Despite recent treatment advances, hemophilia can still have some degree of negative impact on the overall quality of life of adolescents. For instance, parental overprotection and restrictions on daily activities can negatively impact the quality of life of adolescents with hemophilia [53,54]. When patients experience bleeding episodes, these can be very disruptive, requiring them to sometimes withdraw from school and certain social situations. Data from a systematic review and meta-analysis involving children and adolescents from around the world showed school attendance and sports to be positively associated with health-related quality of life, whilst pain due to bleeding was related to a reduction in quality of life for physical health [54]. In the long-term, recurrent joint bleeds can result in permanent joint health issues that can have a lifelong impact on quality of life [39,55]. As prophylaxis can enable patients with hemophilia to achieve a similar quality of life to that of the non-hemophilic population, adolescents with hemophilia should be supported to overcome the barriers facing treatment adherence [9], and by engaging with treatment and learning to manage their condition independently, there is the opportunity for adolescents to take ownership of their future quality of life. 

### 5.3. Sexual Activity

Patients with hemophilia can generally have entirely normal sexual lives [9]. Indeed, some adolescent patients have explained that ‘hemophilia did not influence finding a partner’, nor was it a reason not to have children [17]. However, as hemophilia can exacerbate issues of sexuality commonly associated with adolescence, many adolescents are concerned about how their hemophilia will influence their sexual desirability and sexual performance [56], with psychosocial issues varying on an individual level and potentially being a more important consideration than for previous generations who may have experienced more physical limitations [4]. Some patients also have concerns about reproduction in relation to their genetics and passing on hemophilia to their children [56]. Adolescents with hemophilia have reported muscle and joint bleeding as a result of the physical exertion connected with sexual activity [57,58]. 

As patients start to reach sexual maturity, healthcare professionals ideally should have open conversations with adolescents to help them find strategies to overcome the musculoskeletal complications associated with sexual activity [58], as well as to address any related psychosocial concerns [57]. In addition to being prepared to address the needs of teens directly, healthcare professionals should also provide information and support to parents as well [56] or enable them to access informed advice elsewhere.

### 5.4. Psychosocial Considerations

Some adolescents with hemophilia suffer from low self-esteem, with a lower total Offer Self-Image Questionnaire score reported than for healthy individuals (*p* = 0.007) in a study performed in Turkey [59]. This suggests that adolescents with hemophilia may require psychosocial support during and beyond adolescence [59]. It is recommended that adolescents with hemophilia are supported in learning to self-infuse and understand adherence and bleed management when treated with prophylaxis, thus empowering them to self-manage their own condition [9]. Therefore, these patients can become experts in their own care and provide support to others [22]. Patients can benefit from peer support; however, organizing peer support for this age group and rare disease population can be challenging.

Patients approaching adolescence may also have feelings of uncertainty around transitioning to an adult healthcare service after having attended their pediatric hemophilia center since birth [60,61]; they can feel lost in this new adult environment. Some adolescents with hemophilia feel left behind when they switch from a pediatric center to an adult healthcare service [3]. Therefore, it is important for close cooperation between pediatric and adult healthcare centers to ensure a smooth transition for these patients [4].

Deciding on a career path and where to live are important questions for any adolescent; the imposed and perceived barriers facing those with hemophilia can influence these questions [61]. Some adolescents are concerned about their future life and career [17]. This concern may stem from the fact that some are worried about facing possible discrimination at work [61]. To address this problem, social law counseling and job interview training have been suggested as possible solutions [61]. Furthermore, online resources to help these patients with bleeding disorders make career decisions are available from organizations such as The Haemophilia Society and The National Bleeding Disorders Foundation [62,63]. Parents and caregivers should be directed to information regarding any career options for which individuals with inherited bleeding disorders may not be suited for safety reasons. This will help to inform discussions as patients get older and progress through adolescence, thereby helping to foster realistic expectations.

## 6. Multidisciplinary Approach to Care

It is clear that there is still room for improvement when it comes to managing adolescents with hemophilia. There should be more of a focus on individual adolescents with hemophilia and their needs beyond treatment rather than the needs of their parents [17]. A multidisciplinary approach can support patients as they progress through adolescence. This ensures that patients are equipped to effectively deal with the challenges associated with hemophilia and adolescence and that the treatment benefits experienced during childhood are maintained into adulthood [3].

A multidisciplinary team, including hemophilia experts, nurses, social workers, physiotherapists, dieticians, and psychologists, can lead to a more holistic treatment approach for adolescents with hemophilia [5]. Physiotherapists can have a key, multifaceted role in the multidisciplinary team [64]. Beyond the direct provision of physiotherapy, they can provide information and advice about maintaining healthy joints, including in relation to improving musculature via age- and patient-appropriate physical activity/sport. In addition, physiotherapy can be particularly useful in the multidisciplinary team via the provision of non-pharmacological pain relief [64]. Psychologists and/or social workers could also support adolescents with self-esteem, body image, treatment engagement, and issues relating to school or employment [9,59,65]. However, access to psychosocial support may be challenging due to a lack of resources in hemophilia centers, as treatment may only be available through referral to a psychologist [65]. This multidisciplinary team can also assist with dietary advice, activity coaching, and weight loss [51].

## 7. Communication in the Digital Age

To maintain positive health outcomes whilst transitioning from childhood to adulthood, patients with hemophilia need to be equipped with self-management skills to fully engage with their treatment independently. As digital technology is an integral part of the lives of many current adolescents, online resources could be leveraged to educate adolescents about hemophilia, equipping them with the necessary skills to manage their own condition. Adolescents with hemophilia may be receptive to receiving information via a website or mobile app [66]; indeed, adolescents may prefer this option to in-person communication alone. If this is the case, then healthcare professionals must ensure that they themselves are informed about and proficient in the use of modern technology and that the digital platform used is contemporary. Examples of digital technologies applied to hemophilia include florio HAEMO and florio HAEMO Kids [67], as well as the Web-Accessible Population Pharmacokinetic Service-Hemophilia (WAPPS-Hemo) [68]. Digital tools intended to engage adolescents with hemophilia should be age-appropriate and are likely to vary over time and between regions; national registries may have a role here.

The limited contact by some adolescents with their peers results in a lack of opportunities for experiential learning [69]. For adolescents, COVID-19 led to a decrease in time spent meeting friends face to face, but an increase in communication via digital means [70]. Adolescents with hemophilia may find it beneficial to interact with the online hemophilia community. Online social networking could provide them with an opportunity to learn and share experiences [69]. However, as there is a strong desire to appear like their peers, they may not want to join a patient group and identify as a person with hemophilia. 

Although young people routinely use digital means of communication, in some cases, patients may feel lonely and more isolated by this [71]. These patients may benefit from face-to-face meetings and may find peer support more beneficial in this medium. Healthcare professionals and patient organizations should consider how to make face-to-face meetings and events more attractive to the patient, potentially with the option of allowing friends and family unaffected by hemophilia to attend. Adolescent patients should be encouraged to participate in face-to-face meetings; these meetings can be used to complement the use of digital technology as a means of communication. 

## 8. Final Thoughts

Adolescents with hemophilia are generally able to live a normal life. Although adolescent patients may have concerns about their future, the evolution of therapeutic options and the general improvements in hemophilia management have provided a wide range of benefits, enabling patients to have good joint health and participate in physical activities. However, more work is still needed to address the actual and perceived limitations facing adolescents with hemophilia. For healthcare professionals, it will be both challenging and very rewarding to consider this, as well as the other aspects of management that facilitate support to help adolescents successfully navigate this period of life.

## Figures and Tables

**Table 1:** Summary of suggested solutions to challenges of management of adolescents with hemophilia.

Areas Which Pose Challenges	Suggested Solution(s)
Prevention and management of bleeds	Individualization and personalization of prophylaxis Revisit bleed recognition and early treatment with patients, as well as promoting self-advocacy and self-care management Educate younger cohort of physicians in bleed recognition Deliver continued education throughout adolescence, including recognition and (long-term) consequences of bleeds Facilitate transition by arranging consultations with patients in the absence of parent/caregiver
Treatment adherence	Modification of treatment to achieve patient goals and participate in shared decision-making with the patient and caregivers Early education and training during early adolescence
Joint health and physical activity	Promote participation in physical activity Provide guidance on physical activity-related queries from patients and on the preparation required before participating in physical activity
Other health-related issues	Provide support around barriers related to treatment adherence Have open conversations with patients to help them find strategies to overcome the musculoskeletal complications and psychosocial concerns around sexual activity, as well as support parents/caregivers Provide psychosocial support throughout adolescence Close cooperation of pediatric and adult healthcare centers to aid transition
Multidisciplinary approach	A multidisciplinary approach can support the challenges associated with hemophilia and adolescence
Communication in the digital age	Utilize digital technology to communicate with patients, but also encourage face-to-face meetings Remain informed and proficient in the use of modern technology, and ensure that the digital platform used to communicate with patients is contemporary Consider how to make face-to-face meetings and events more attractive to the patient

## Data Availability

This article contains no new data; data sharing is not applicable.
